# Thrombotic Microangiopathy in the Renal Allograft: Results of the TMA Banff Working Group Consensus on Pathologic Diagnostic Criteria

**DOI:** 10.3389/ti.2023.11590

**Published:** 2023-08-23

**Authors:** Marjan Afrouzian, Nicolas Kozakowski, Helen Liapis, Verena Broecker, Luon Truong, Carmen Avila-Casado, Heinz Regele, Surya Seshan, Josephine M. Ambruzs, Alton Brad Farris, David Buob, Praveen N. Chander, Lukman Cheraghvandi, Marian C. Clahsen-van Groningen, Stanley de Almeida Araujo, Dilek Ertoy Baydar, Mark Formby, Danica Galesic Ljubanovic, Loren Herrera Hernandez, Eva Honsova, Nasreen Mohamed, Yasemin Ozluk, Marion Rabant, Virginie Royal, Heather L. Stevenson, Maria Fernanda Toniolo, Diana Taheri

**Affiliations:** ^1^ Department of Pathology, John Sealy School of Medicine, University of Texas Medical Branch at Galveston, Galveston, TX, United States; ^2^ Department of Pathology, Medical University of Vienna, Vienna, Austria; ^3^ Nephrology Center, Ludwig Maximilian University of Munich, Munich, Germany; ^4^ Department of Pathology, Sahlgrenska University Hospital, Gothenburg, Sweden; ^5^ Department of Pathology and Genomic Medicine, The Houston Methodist Hospital, Houston, TX, United States; ^6^ Laboratory Medicine Program, Toronto General Hospital, University Health Network (UHN), Toronto, ON, Canada; ^7^ Department of Pathology and Laboratory Medicine, Weill Cornell Medicine, New York, NY, United States; ^8^ Arkana Laboratories, Little Rock, AR, United States; ^9^ Department of Pathology and Laboratory Medicine, Emory University, Atlanta, GA, United States; ^10^ Department of Pathology, Université de Sorbonne, Assistance Publique—Hôpitaux de Paris, Hôpital Tenon, Paris, France; ^11^ New York Medical College, Valhalla, NY, United States; ^12^ Department of Pathology and Immunology, Baylor College of Medicine, Houston, TX, United States; ^13^ Department of Pathology and Clinical Bioinformatics, Erasmus University Center Rotterdam, Rotterdam, Netherlands; ^14^ Institute of Experimental Medicine and Systems Biology, RWTH Aachen University, Aachen, Germany; ^15^ Departamento de Patologia Geral, Instituto de Ciências Biológicas, Universidade Federal de Minas Gerais, Belo Horizonte, Brazil; ^16^ Department of Pathology, Koç University School of Medicine, Istanbul, Türkiye; ^17^ Department of Anatomical Pathology, NSW Health Pathology, Callaghan, NSW, Australia; ^18^ School of Medicine and Public Health, College of Health, Medicine and Wellbeing, The University of Newcastle, Callaghan, NSW, Australia; ^19^ Department of Pathology, School of Medicine, University of Zagreb, Zagreb, Croatia; ^20^ Department of Pathology and Laboratory Medicine, Mayo Clinic, Rochester, MN, United States; ^21^ AeskuLab Pathology and Department of Pathology, Charles University, Prague, Czechia; ^22^ Department of Pathology and Laboratory Medicine, King Fahad Specialist Hospital-Dammam, Dammam, Saudi Arabia; ^23^ Department of Pathology, Istanbul Faculty of Medicine, Istanbul University, Istanbul, Türkiye; ^24^ Department of Pathology, Necker-Enfants Malades Hospital, Université Paris Cité, Paris, France; ^25^ Department of Pathology, Maisonneuve-Rosemont Hospital, University of Montreal, Montreal, QC, Canada; ^26^ Kidney Pancreas Transplantation, Instituto de Nefrología-Nephrology, Buenos Aires, Argentina; ^27^ Department of Pathology, Isfahan Kidney Diseases Research Center, Isfahan University of Medical Sciences, Isfahan, Iran; ^28^ Urology Research Center, Sina Hospital, Tehran University of Medical Sciences, Tehran, Iran

**Keywords:** thrombotic microangiopathy, kidney, transplant, pathology criteria, Delphi, Banff

## Abstract

The Banff community summoned the TMA Banff Working Group to develop minimum diagnostic criteria (MDC) and recommendations for renal transplant TMA (Tx-TMA) diagnosis, which currently lacks standardized criteria. Using the Delphi method for consensus generation, 23 nephropathologists (panelists) with >3 years of diagnostic experience with Tx-TMA were asked to list light, immunofluorescence, and electron microscopic, clinical and laboratory criteria and differential diagnoses for Tx-TMA. Delphi was modified to include 2 validations rounds with histological evaluation of whole slide images of 37 transplant biopsies (28 TMA and 9 non-TMA). Starting with 338 criteria in R1, MDC were narrowed down to 24 in R8 generating 18 pathological, 2 clinical, 4 laboratory criteria, and 8 differential diagnoses. The panelists reached a good level of agreement (70%) on 76% of the validated cases. For the first time in Banff classification, Delphi was used to reach consensus on MDC for Tx-TMA. Phase I of the study (pathology phase) will be used as a model for Phase II (nephrology phase) for consensus regarding clinical and laboratory criteria. Eventually in Phase III (consensus of the consensus groups) and the final MDC for Tx-TMA will be reported to the transplantation community.

## Graphical Abstract

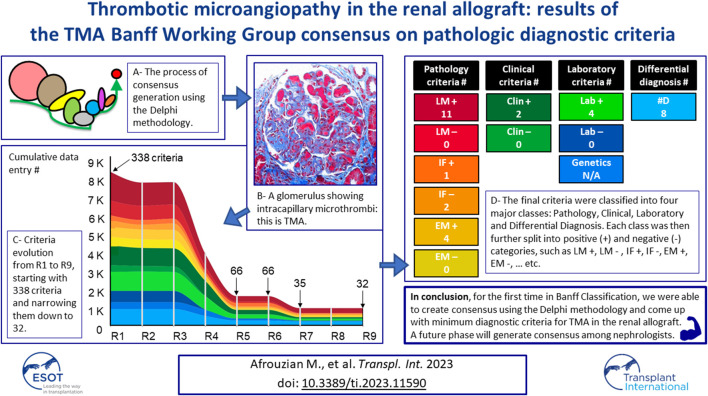

## Introduction

Transplant thrombotic microangiopathy (Tx-TMA) is caused by endothelial injury which is hallmarked by thrombotic occlusion of small vessels resulting in often clinically unexpected allograft failure [1, 2]. Immunologic, genetic, hematologic disorders and drugs may trigger the disorder [1, 3]. A transplant kidney biopsy is performed for definitive diagnosis [4].

The histopathologic diagnosis of Tx-TMA relies on the subjective interpretation of a multitude of histopathologic findings of which thrombi is the major one, but varies in extent and frequency, and depends on its acute or chronic character, and, finally, on the pathologist. There is a long list of morphologies making the diagnosis challenging and often delaying initiation of targeted therapy. The Banff TMA working group (WG) was formed in 2016 under the auspices of the Banff Foundation for Allograft Pathology, with the aim of standardizing TMA diagnostic criteria and coming up with recommendations [5]. A survey circulated in January 2016 among the WG participants, showed considerable heterogeneity among nephropathologists, using a multitude of known TMA features (as mentioned above) with vague or subjective definitions. Therefore, the first aim of the WG was to provide the Banff community with a standardized set of minimum diagnostic criteria for Tx-TMA. A secondary ambition which was identified during the study was to investigate specific lesions that could potentially determine specific etiologies of Tx-TMA. Diagnosis of TMA in the renal allograft, is not merely a morphologic exercise; clinical and laboratory information is crucial for diagnosis. The Delphi approach was considered by the co-chairs as a suitable method to generate consensus, among an expert panel [6–9].

## Materials and Methods

A detailed description of the materials and methods used in this project including establishing a steering committee, literature review, definition of a panelist, the role of the facilitator, and the process and sequences of events during Delphi rounds is presented in paper 1 [10]. Herewith in paper 2, the authors describe those specific aspects of the materials and methods that are related to pathology.

In the preliminary round, R0, the facilitator asked several questions related to the diagnosis of Tx-TMA and requested the panelists to send their areas of difficulty with Tx-TMA diagnosis in free text. The questions are listed in Supplementary Table S1.

### Cut-Offs

At the end of each R and after receipt of panelists’ responses and data analysis, the cut-off for that R was chosen by the facilitator. It is important to emphasize that the Delphi methodology allows the facilitator to arbitrarily set cut-offs for Rs. This is to allow the facilitator to set the cut-off at a level where redundancies can be eliminated, but the most important information could be retained for the next R. In our study, a cut-off of 80% was set for all Rs, except for R4 and R5. To make sure that no important criterion is dropped for the next R, the cut-off for these two Rs was set at 60%, as a cut-off of 80% would have eliminated well-known TMA lesions, such as presence of double contours.

### Pathological Validation of the Criteria

The original Delphi method used in other disciplines or in earlier pathology manuscripts did not contain a histology-based validation round. In this study, we designed a modified version of Delphi to adapt the methodology to the needs of our study, which was a pathology project, where the results of the rounds needed to be validated using real-life cases. Therefore, at the beginning of the study, the facilitator asked the panelists to submit transplant kidney biopsy (TxBx) cases from their institutional collection. A total of 37 cases of TxBx was collected and shipped to the facilitator (MA) at the Department of Pathology. For each case, 2–3 micron-thick paraffin-embedded sections, stained with hematoxylin & eosin (H&E), periodic-acid-Schiff (PAS), Masson’s trichrome (TCR) and Jones silver or periodic-acid-methenamine-silver (PAMS) stains were submitted. IF and immunohistochemistry (IHC) including C4d staining, as well as EM findings were provided in free text. Only some cases were supplemented by EM images. Slides were de-identified and scanned at ×400 using an Aperio scanner at the University of Toronto. Central review of the cases was performed by the steering committee before circulating the cases among the panelists.

### The Cases

Histological evaluation was included in the Delphi process during rounds R6 and R7, where 66 criteria (56 pathological, clinical and laboratory criteria and 10 differential diagnoses) were validated against 37 real-life cases. The panelists were asked to list the criteria they used to make their diagnosis on each case. The cases validated in this study were composed of TMA cases (*n* = 28) and non-TMA cases or look-alikes (*n* = 9), displayed in Supplementary Table S2. The original diagnosis of the 37 validated anonymized cases along with the patients’ demographics reflected a random selection of real-life situations encountered by our panelists in their practice. Each case was accompanied by a short clinical history, relevant laboratory information available at the time of biopsy. The co-chairs also received the original pathology report and diagnosis, and information regarding treatment and outcome, which were not shared with the panelists.

### Percentage Agreement (%A) and Percentage Agreement Levels (%AL)

%A shows agreement amongst the panelists concerning a diagnosis or criterion. Moreover, we computed the level of agreement as the number of cases falling into a %AL. For example, a 97–100%AL was the level on which 97%–100% of the panelists agreed on the same diagnosis on X number of cases. Further, a %AL was considered: 0–40 = poor; 41–60 = fair; 61–80 = good; 81–96 = excellent and 97–100 = total.

### Statistics

All statistical modeling were performed using SAS, version 9.4 (SAS, Inc., Cary, NC). Details on the statistics are published in paper 1 [10]. Some figures were drawn using the open source data visualization tool RAWGraphs [11].

Of note, this study used a retrospective collection of cases to validate criteria resulting from the consensus and was not designed to measure outcome, therapy, or intervention.

## Results

### Pathological Criteria

Table 1 lists the six pathological categories and their related criteria. A total of 18 pathological criteria (16 positive or 2 negative) were obtained at the end of R7.

**TABLE 1:** Pathological criteria classified in 6 categories and panelists’ percentage of agreement (%A) for each criterion.

Category 1	LM + criteria	%A
1	1A. bloodless, dilated, congested glomerular capillaries	54
2	1B. fibrin thrombi in arterioles/small arteries ± fibrinoid change	100
3	1C. fibrin thrombi in glomerular capillaries/hilum	100
4	1D. arterial or arteriolar intimal edema/mucoid changes	95
5	1E. glomerular endothelial swelling (acute lesion)	73
6	1F. mesangiolysis (acute lesion)	82
7	1G. double contours (chronic lesion)	59
8	1H. platelet thrombi in glomerular capillaries (CD61)	50
9	1I. fragmented/extravasated RBCs	50
10	1J. onion skin changes (chronic lesion)	41
11	1K. collapsed capillaries	18

Category 2	LM **–** **criteria**	
0	There is no LM finding that can help ruling out TMA	73

Category 3	IF + criteria	
1	3A. glomerular intraluminal staining with fibrin-related antigens	91

Category 4	IF **–** **criteria**	
1	4A. C4d positivity in peritubular capillaries (favoring AMR vs. TMA)	82
2	4B. presence of immune complexes	77

Category 5	EM + criteria	
1	5A. sub-endothelial widening/rarefaction + accumulation of “fluff”	91
2	5B. fibrin tactoids in the lumen/widened sub-endothelial space (glomerular or vascular)	91
3	5C. glomerular endothelial swelling, loss of/decreased fenestration (acute lesion)	86
4	5D. GBM duplication/lamination/multilayering with mesangial (or mesangial cell) interposition (chronic lesion)	86

Category 6	EM **–** **criteria**	
0	There is no EM lesion that can help you rule out TMA	82

The following lists the pathological criteria:

- *11 LM+ criteria* including presence of bloodless, dilated, congested glomerular capillaries; fibrin thrombi in arterioles/small arteries ± fibrinoid change; fibrin thrombi in glomerular capillaries/hilum; arterial or arteriolar intimal edema/mucoid changes; glomerular endothelial swelling (acute lesion); mesangiolysis (acute lesion); double contours (chronic lesion); platelet thrombi in glomerular capillaries; fragmented/extravasated red blood cells (RBCs); onion skin changes (chronic lesion); collapsed capillaries.
- *1 IF+ criterion* including presence of glomerular intraluminal staining with fibrin-related antigens.
- *2 IF- criteria* including C4d-positivity in peritubular capillaries (favoring AMR vs. TMA), and presence of immune complexes.
- *4 EM+ criteria* including sub-endothelial widening/rarefaction + accumulation of “fluff”; fibrin tactoids in the lumen/widened sub-endothelial space (glomerular or vascular); glomerular endothelial swelling, loss of/decreased fenestration (acute lesion); GBM duplication/lamination/multilayering with mesangial (or mesangial cell) interposition (chronic lesion).

During this process, the panelists put an emphasis on the temporal character of the lesions, for instance, intracapillary thrombi reflecting acute and/or sub-acute Tx-TMA, while double contours, representing chronic Tx-TMA. Of note, acute, sub-acute and chronic TMA were considered as phenomena that can be present simultaneously.

### Clinical Criteria

The 2 Clin+ criteria shown in Table 2 included pregnancy/post-partum/history of pre-eclampsia/eclampsia HELLP syndrome and past history of TMA/HUS/aHUS/TTP.

**TABLE 2:** Clinical and laboratory criteria and panelists’ percentage of agreement (%A) for each criterion.

Category 7	Clin + criteria	%A
1	7A. pregnancy/post-partum/history of pre-eclampsia/eclampsia/HELLP syndrome	91
2	7B. past history of TMA/HUS/aHUS/TTP	91

Category 8	Clin - criteria	
0	There is no clinical info that can help you ruling out TMA	77

Category 9	Lab + criteria	
1	9A. elevated LDH	91
2	9B. low haptoglobin levels (in the absence of history of recent transfusion)	91
3	9C. dropping hematocrit/anemia/hemolytic anemia	91
4	9D. thrombocytopenia	91

Category 10	Lab - criteria	
0	No criterion was retained	00

Category 11	Gen criteria were not assessed	
0	none	00

### Laboratory Criteria

Table 2 also shows the results on the laboratory criteria.

The 4 Lab+ criteria included elevated LDH, low haptoglobin levels (in the absence of history of recent transfusion), dropping hematocrit/anemia/hemolytic anemia and thrombocytopenia. Two Lab-criteria were dropped because of insufficient votes (<20%): absence of donor ABO-incompatibility and absence of proteinuria.

### Differential Diagnoses

Table 3 presents the eight differential diagnoses most used during the validation of the 37 cases. They were entertained during the two validation Rs and included thrombotic thrombocytopenic purpura (TTP)/acquired HUS/atypical HUS (aHUS); donor-related TMA: observed in the donor in the first week/first month post Tx; chronic Tx glomerulopathy; disseminated intravascular coagulation (DIC); acute or chronic non-TMA-related ABMR (NT-ABMR); anti-phospholipid syndrome; immune complex-mediated glomerulonephritis (GN) including *de novo* or recurrent membranoproliferative GN, IgA nephropathy (IgAN), lupus nephritis (LN), post-infectious GN and accelerated hypertension.

**TABLE 3:** Differential diagnoses.

Category 12	#D	%A
1	12A. TTP/Acquired HUS/aHUS	82
2	12B. donor-related TMA: observed in the donor in the first week/first month post Tx	86
3	12C. chronic TX glomerulopathy	82
4	12D. DIC	73
5	12E. acute or chronic NT-ABMR	77
6	12F. anti-phospholipid syndrome	59
7	12G. immune complex-mediated GN (*de novo* or recurrent, MPGN, IgAN, LN, post-infectious GN)	45
8	12H. accelerated hypertension	41

### Definitions

At the end R8, the need to generate consensus regarding morphological definition of key lesions was recognized. In R9, eight criteria were defined. Table 4 lists the definition of 4 LM and 4 EM criteria on which consensus was obtained among the panelists.

**TABLE 4:** Definitions for selected light and electron microscopy lesions.

Light microscopy	
1A. bloodless, dilated, congested glomerulus	Ischemic wrinkling (=“deflation”, = “ghost glomerulus”, = “implosion”) of capillary loops mostly devoid of RBCs, ± enlarged endothelial cells, ± luminal occlusion, ± thickened GBM appearing less dense on Jones silver stain (=sub-endothelial accumulation by EM)
1D. arterial or arteriolar intimal edema/mucoid change	Arterial or arteriolar intimal expansion or widening with edema and accumulation of basophilic material (mucoid/mucinous/myxoid change) ± luminal narrowing
1F. mesangiolysis (acute lesion)	Poorly stained (=“dissolution”) widened mesangium, ± dilated capillary loops or microaneurysms, ± loss or degenerative changes of mesangial cells
1I. Fragmented, extravasated RBC	Arterial or arteriolar intramural fragmented RBCs

Electron microscopy	
5A. sub-endothelial widening/rarefaction + accumulation of “fluff”	Sub-endothelial loose and finely granular, flocculent electron lucent material (“fluff”) ± fibrin* tactoids in glomeruli, arteries or arterioles. (* = fibrin in the lumen can be caused by other diseases)
5B. fibrin tactoids in the lumen/widened sub-endothelial space (glomerular or vascular)	Spiculated, needle- or spindle-shaped tactoid material, at times striated
5C. glomerular endothelial swelling, loss of/decreased fenestration (acute lesion)	EC loss of fenestration with cytoplasmic vacuolization
5D. GBM duplication/lamination/multilayering with mesangial (or mesangial cell) interposition (chronic lesion)	Mesangial cell or matrix interposition indicating chronic lesion*. (*: presence of fluff indicates acute lesion)

### Criteria Evolution During Nine Rounds

**FIGURE 1:**
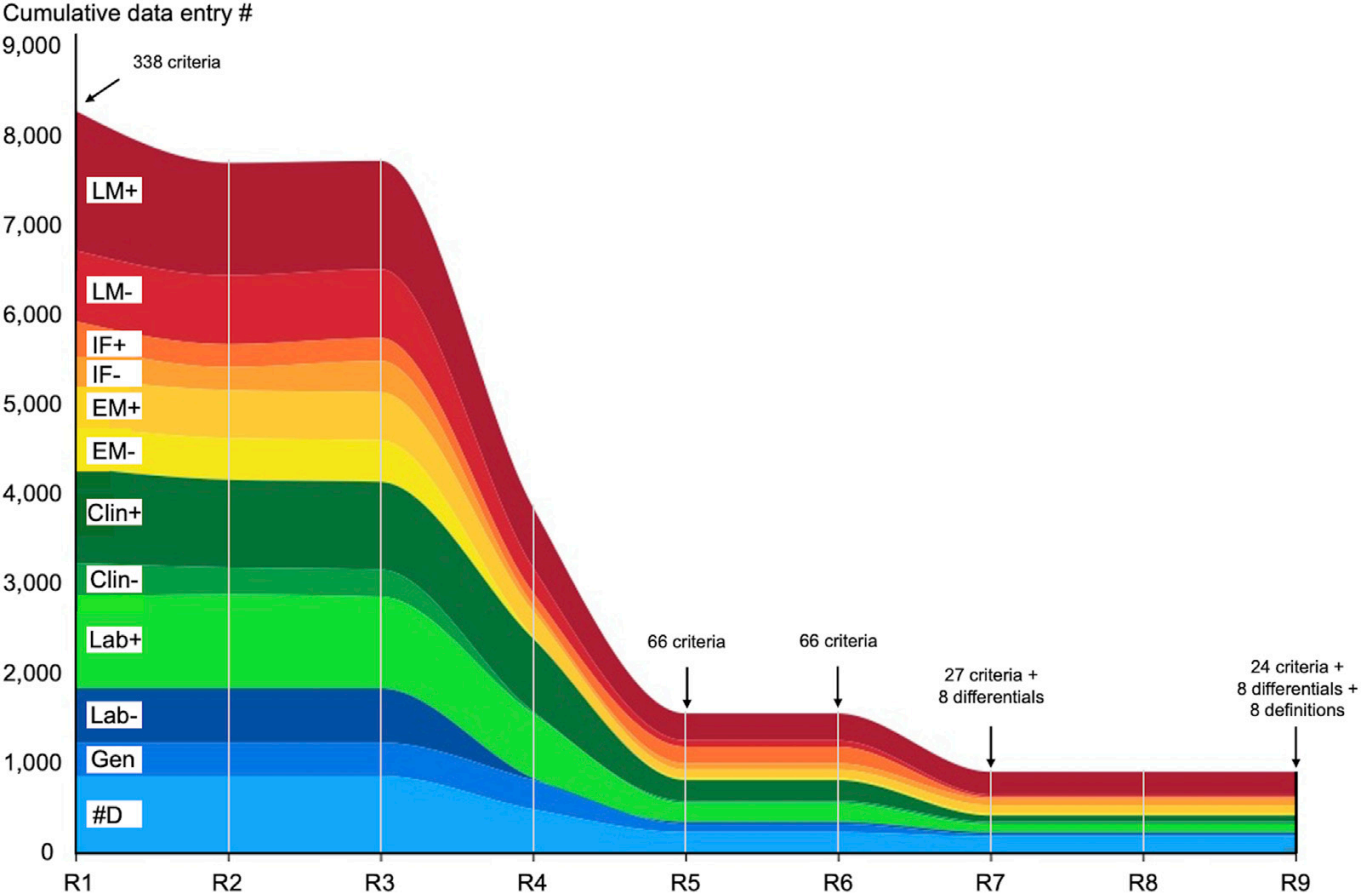
Criteria evolution during eight rounds of Delphi. The X-axis represents each R and the Y-axis the cumulative number of data entries. R1 started with 338 criteria (arrow) which were narrowed down to 66 in R5 and remained 66 in R6. The criteria were further narrowed down to 35 (comprising eight differential diagnosis) in R7. R6 and R7 were two validation rounds and R9 was the control round also called the definition round. Abbreviations: Clin-, clinical data negative; Clin+, clinical data positive; #D, differential diagnosis; EM-, electron microscopy negative; EM+, electron microscopy positive; IF-, immunofluorescence microscopy negative; IF+, immunofluorescence microscopy positive; Lab-, laboratory negative; Lab+, laboratory positive; LM-, light microscopy negative; LM+, light microscopy positive.

Figure 1 shows criteria evolution from R1 to R9. A detailed explanation of the evolution of the criteria is reported in the result and discussion sections of paper 1 [10].

Basically, starting with 338 criteria obtained at the end of R1, the facilitator was able to narrow them down to a final number of 24 criteria and 8 differential diagnoses at the end of the study.

### Quality of the Panelists’ Agreement

The panelists’ diagnostic performance on the 37 cases computed at 61–80%AL, 81–96%AL and 97–100%AL is shown in Table 5: The 61–80%AL column shows that up to 80% of the panelists agreed on 83.78% of cases (31/37) which represents a “good” level of agreement. The 81–96%AL column shows that up to 96% of panelists agreed on 54.05% of the cases (20/37) which is considered an “excellent” level of agreement on more than the half of the cases. Total agreement or 97–100%AL between the panelists was obtained on 10.81% of cases (4/37). In each column, those cases marked with (-) did not reach the %AL indicated for that column. It is worth noting that regarding choosing between a diagnosis of Tx-TMA vs. no TMA, on six cases (16.21%), the panelists’ opinions were split (12 vs. 11). Agreement on these six cases was therefore judged as “equivocal”. A more detailed information about the cases and their respective %AL is provided in Table 5.

**TABLE 5:** Original diagnoses on the 37 cases, panelists’ responses, percentage agreement (%A) and percentage agreement levels (%AL).

Case #	Original diagnoses	Panelist responses		(%A)		(%AL)		
		TMA	No TMA	TMA	No TMA	61–80 %AL in 31/37 cases (83.8%)	81–96 %AL in 20/37 cases (54.1%)	97–100 %AL in 4/37 cases (10.8%)
1	TMA (diffuse)	19	4	83	17	X	X	—
2	TMA (focal) + AMR	22	1	96	4	X	X	—
3	TMA (acute & chronic)	23	0	100	0	X	X	X
4	TMA (classical case)	22	1	96	4	X	X	—
5	TMA (classical case)	22	1	96	4	X	X	—
6	TMA (early)	11	12	48	52	—	—	—
7	TMA found on EM only	8	15	35	65	X	—	—
8	TMA found on EM only	4	19	17	83	X	X	—
9	TMA (classical case)	22	1	96	4	X	X	—
10	AMR + TMA	12	11	52	48	—	—	—
11	TMA (classical case)	19	4	83	17	X	X	—
12	No TMA (suspicious for AMR)	7	16	30	70	X	—	—
13	No TMA (TCMR +C4d-neg AMR)	5	18	22	78	X	—	—
14	Subtle TMA + CNI tox	14	9	61	39	—	—	—
15	TMA (classical case)	20	3	87	13	X	X	—
16	TMA (classical case)	17	6	74	26	X	—	—
17	TMA with rare thrombi	19	4	83	17	X	X	—
18	TMA with small thrombi	5	18	22	78	X	—	—
19	No TMA (GN with deposits)	4	19	17	83	X	X	—
20	TMA (acute and chronic)	22	1	96	4	X	X	—
21	TMA (acute and chronic)	21	2	91	9	X	X	—
22	TMA + Nephrosclerosis	18	5	78	22	X	—	—
23	No TMA (chronic AMR + TG + weak C4d+)	10	13	43	57	—	—	—
24	No TMA (chronic AMR + TG + weak C4d+)	6	17	26	74	X	—	—
25	TMA (classical case)	22	1	96	4	X	X	—
26	TMA (classical case)	21	2	91	9	X	—	—
27	TMA + Hypertensive arteriopathy	21	2	91	9	X	—	—
28	TMA (classical case)	23	0	100	0	X	X	X
29	TCMR	5	18	22	79	X	—	—
30	TMA (focal) + AMR	12	11	52	48	—	—	—
31	TMA (classical case)	21	2	91	9	X	X	—
32	No TMA	12	11	52	48	—	—	—
33	TMA (classical case)	23	0	100	0	X	X	X
34	No TMA (rec. MPGN)	14	19	42	58	X	—	—
35	No TMA (rec. IgA nephropathy)	2	21	9	91	X	X	—
36	TMA (classical case)	23	0	100	0	X	X	X
37	TMA + AMR	21	2	91	9	X	X	—

R8 was originally planned to produce major and minor criteria according to the panelists’ ranking; however, after examination of the results, the facilitator decided that future validation studies are needed to develop the concept of major/minor criteria.

### Literature Review

An exhaustive literature search and review (12–27) regarding the incidence of the selected lesions of Tx-TMA obtained at the end of R8 revealed that there is a lack of systematic reporting on the incidence of 12 pathological lesions/criteria obtained in the current study. Supplementary Table S3 summarizes the result of the literature review [12–26].

## Discussion

### TMA in the Native and the Transplanted Kidney: Similarities and Differences

TMA in the native kidney shares many morphological features with TMA in the transplanted kidney. They both are caused by endothelial cell injury, and presence of intravascular thrombi, and especially when the lesions are diffuse, they are strong diagnostic tools for the pathologist. However, similarities between the two conditions stop at the morphological level as a transplanted organ is involved with and targeted by many factors that a native organ is not. TMA in the native kidney: 1. is typically part of a larger picture and one of the manifestations of a systemic disease such as Hemolytic Uremic Syndrome (HUS); 2. is associated with laboratory indicators of microvascular thrombosis, such as thrombocytopenia, elevated LDH and decreased haptoglobin; 3. is usually the only main finding in the biopsy; 4. is often the manifestation of a single disease, for example, systemic sclerosis or systemic lupus erythematosus. On the other hand, Tx-TMA often: 1. presents as localized TMA (L-TMA or renal TMA), and not as part of a systemic disease. While recurrent disease is the cause of a small proportion of Tx-TMAs, most transplant L-TMAs are *de novo* [27]; 2. lacks the laboratory indicators of microvascular thrombosis such as thrombocytopenia, presence of schistocytes, elevated LDH; 3. is difficult to diagnose as there are many confounding factors, such as antibody-mediated rejection (C4d-positive or C4d-negative), T cell-mediated rejection, drug toxicity, and recurrence of the pre-existing disease that blurs the picture for both clinical and pathological diagnosis. Therefore, while endothelial injury is central to the pathogenesis in both renal native and allograft TMA leading to similar lesions in the glomerulus and renal vasculature, diagnosis of Tx-TMA involves a different mindset, algorithm, and differential diagnosis, and sometimes, different criteria.

### Literature Review

Up-to-date and to the authors’ knowledge, there is no study dealing with the standardization of diagnostic criteria for Tx-TMA (Supplementary Table S3). The paper published by Haas et al [28], addresses the diagnostic criteria for TMA, however, only touches TMA in the native kidney and TMA in the renal allograft is not approached. Most scientific literature does not provide a detailed description of Tx-TMA-associated lesions, including the pathological criteria for which our study reached a consensus. Thus, our study fills this gap and provides, for the first time, diagnostic criteria as prerequisite for further comparative studies.

### The TMA BWG Mandates: The Why and the What

As the results of the 2016 Banff TMA WG clearly showed, nephropathologists use many different criteria/lesions to diagnose Tx-TMA. The TMA BWG was formed with specific objectives and goals to standardize the existing biopsy lesions, retrospectively [29]. The goals of the TMA BWG, according to the Banff 2017 meeting report were to: “1- establish uniform diagnostic criteria for Tx-TMA; 2- determine the frequency with which TMA occurs in renal allograft biopsy; and 3- determine if there are specific features of TMA in renal allografts that help resolve the differential diagnosis of Tx-TMA when the cause is not readily apparent from clinical history, DSA/C4d, etc…”

The authors achieved the first goal in 5 years and generated consensus among Banff participants regarding establishing a list of diagnostic criteria. The second goal was accomplished by reviewing the current literature: the authors unveiled the lack of data on the incidence of the Tx-TMA lesions Tx-TMA lesions identified through this Delphi study. The third goal could not be achieved entirely as further input from nephrologists will be needed to finalize the clinical and laboratory criteria. The Phase II of the study with nephrologists is currently in progress and will address the third goal.

### Novelty of the Study: Introducing Delphi to the Banff Classification

Since 1991 and for the past 30 years, the Banff Classification on Allograft Pathology group used the NIH model of consensus generation as a tool to define transplant-related pathological lesions. This required resources for travelling and live meetings amongst expert pathologists, nephrologists, and transplant surgeons. The debates resulted in recommendations known as Banff criteria, which were proposed to the transplantation community, and applied for patient management, following rigorous validation studies. Although Delphi by itself is not a new methodology, it solves many of the inconveniences of the use of the NIH consensus format within the Banff community: anonymous yet democratic approach of consensus generation; first-time introduction of digital pathology to Delphi for case validation; and dramatic reduction of the costs of a Banff-related process. The total cost of the study was below US$20,000.00. As no travelling was required, in the era of global warming and the COVID pandemic, this methodology suggests a new approach for consensus generation to the Banff community. In the joint paper of our working group describing the Delphi process, readers will find why they should choose one method over the other [10].

It took 5 years to complete this study and come up with 24 criteria and 8 differential diagnoses. The time may seem long, however, if compared to allograft rejection introduced in Banff in 1991 which took 20 years for the Banff community, to reach consensus on final diagnostic criteria, this appears a speedy process. An example is the glomerulitis lesion (g lesion) which was introduced in Banff in 1993 [30]. Although the criteria were introduced at that time, their definition and application evolved continuously throughout the years, discussions continued for years regarding threshold for number of glomerular leukocytes, the degree of endothelial cell enlargement/capillary luminal occlusion or even the exact application of the g score [31, 32]. The consensus for these lesions took 18 years, 9 Banff conferences held in multiple locations including Banff/Canada, Aberdeen/Scotland, La Coruna/Spain, Edmonton/Canada, and Paris/France to come up with final diagnostic criteria on glomerulitis. In comparison, our Delphi study started with 338 suggestions, involved 23 panelists (all nephropathologists) and 4 nephropathologists who conducted the study. The study was completed in 5 years (despite the pandemic turmoil), with significantly smaller budget. The low cost of the Delphi method is not specific to this study and is a known advantage of Delphi.

### Panelists’ Performance

Panelists’ performance from a statistical point of view, is briefly discussed in paper 1 [10]. In the current paper, the authors would like to put an emphasis on the impact that the complexity of TX-TMA cases have on the pathologists’ performance.

Light, immunofluorescence and electron microscopy criteria listed in Table 1 are the results of nine rounds of survey. The listed criteria do not represent any new lesions and every pathologist dealing with Tx-TMA uses some of them during his/her practice. This list is basically a guideline on the most important lesions that need to be considered when dealing with Tx-TMA. Some aspects of Tx-TMA also will need to be tested by additional studies with prediction analysis. For example, the distinction between chronic and acute lesions of Tx-TMA seems to be important, as they are manifested by different microscopic lesions. The presence of acute TMA lesions generally means the patient has an on-going treatable condition, while chronic TMA lesions generally mean the patient has potentially irreversible damages in the renal allograft. The usefulness of distinguishing chronic from acute TMA therefore could be the subject of such prediction analysis.

At this point the authors draw the reader’s attention to an important point: The “subjects” in this Delphi study are neither the criteria nor the real-life cases that were validated. The “subjects” are “the panelists.” Therefore, statistics usually expected from an NIH-type study such as adequacy of the sample size or number of validated cases, and reporting of *p*-values and ICCs related to criteria, should not be expected from this Delphi study. Only %A and %AL which reflect subjects’ or panelists’ performance can be reported. This is one of the main differences between Delphi and NIH-type consensus methods. Delphi evaluates performance at different agreement levels, not the criteria nor the cases. Therefore, the final results will not be presented with *p*-values or ICC but as total, excellent, good, fair or poor agreement levels.

### Supporting Clinical and Laboratory Criteria

For the pathological diagnosis of Tx-TMA, the clinical situations such as arterial hypertension, acute renal or multi-system organ failure were deemed unnecessary, as well as laboratory items such as donor specific antibodies (DSA), positive crossmatch, low complement levels or high serum levels of CNIs, since the panelists believed none of these criteria can stand alone.

Despite the fact that clinical and laboratory information are essential for renal biopsy interpretation, consensus was reached on only a few criteria. Early on during the Delphi process, our renal transplant pathology expert panelists suggested and listed both therapeutic agents (for example, Tacrolimus or mTOR inhibitors) and complement-related disorders as items that could be considered in the final list of diagnostic criteria. However, as the list was narrowed down to reflect minimum diagnostic criteria, these items were eliminated by consensus. Additionally, the majority of the 37 cases shared by the panelists and validated, did not have any initial information about complement factors, as it happens in real-life situation and early in the course of diagnosing a case of Tx-TMA. Therefore, these items are not listed in this phase of the study. Importantly, this information is not lost, and being entertained in Phase II (as mentioned above) by the nephrologists.

This is consistent with the difficulty that nephrologists and nephropathologists have in diagnosing Tx-TMA. Even though in the pathology phase (Phase I) these criteria were agreed on, they will need to be approved by the nephrologists in Phase II. They are, therefore, not final.

### Emergence of Areas of Controversy

After reviewing the panelists’ responses on the 37 cases, the most common confounding factor for pathology diagnosis of Tx-TMA emerged: ABMR. It became a source of considerable intellectual conflict every time a case that had a clinical, laboratory (C4d or DSA results) or morphological hint of ABMR was encountered by the panelists. To explain the magnitude of the problem: one of the most challenging questions for our panelists was whether ABMR is in the differential diagnosis list of Tx-TMA or is causing Tx-TMA? Therefore, ABMR and its attributes were mentioned both as negative criteria when the panelists were trying to rule out Tx-TMA, and at the same time as criteria for diagnosis of Tx-TMA. The authors believe this area of conflict needs to be addressed by the Banff community, requiring further research and debate, and is out of the scope of this paper.

### Strengths and Weaknesses of the Study

Comparisons between the Delphi method and other consensus generation tools, including the NIH-type method, have been discussed in detail in the literature [8]. For our study, the reasons why we chose the Delphi methodology, which we consider a strength, were multiple: its anonymous aspect, its capacity to generate consensus among many participants, on numerous items, and in a short period of time, as well as its huge advantage on cost-effectiveness. The Delphi methodology has recently been used in surgical pathology [33, 34], however, this is the first time that the method is being used in the Banff classification group. Leading to rapid and inexpensive consensus, this process could represent a precedent in consensus generation within the Banff community. One of the advantages of Delphi is the flexibility that the facilitator has in designing the rounds. However, our study went beyond a general survey on opinions related to Tx-TMA and included histological evaluation of real-life cases within consensus generation to define diagnostic lesions. Online surveys allowed to respect our initial wish for anonymous responses.

The lack of accepted criteria that would play the role of gold standard in the diagnosis of the 37 cases not only was one of the main hurdles of this study, but also the main motivation behind initiating this work. During the two validation Rs, to circumvent this obstacle, it was decided to adhere to the original diagnosis provided by the panelist/expert who had submitted the cases.

Perhaps a further caveat of the study is the lack of correlation with treatment and outcome.

Despite the above-mentioned weaknesses, this study represents a significant step forward to tackle the pathology issues associated with Tx-TMA. A second Delphi study, with the collaboration of over 30 nephrologists, is currently ongoing.

## Conclusion

The current work is a starting point in the process of diagnosing renal Tx-TMA. The TMA BWG looked at Tx-TMA from many different perspectives including its patterns of appearance (systemic versus localized), temporal occurrence (acute versus chronic), the difficulties pathologists face in identifying some of its lesions by LM, relationship between Tx-TMA and ABMR, and other potentially confounding conditions, and finally, the multitude of its mimickers (differential diagnoses). The authors generated consensus on 24 criteria, providing a list of differential diagnoses and identifying areas of diagnostic difficulty. While this realization undoubtedly conveys valuable recommendations for nephropathologists involved in the management of patients with Tx-TMA, its satisfactory implementation will require attentive validation and refinement, starting with consensus generation among nephrologists, who will fortify the clinical and laboratory criteria. Once Phase II and Phase III are completed, this study may serve as a baseline for diagnosing Tx-TMA, and Delphi be considered a useful methodology facilitating the process of consensus generation within the transplantation community.

## Data Availability

The original contributions presented in the study are included in the article/Supplementary Material, further inquiries can be directed to the corresponding author.

## Abbreviations

ABMR, antibody-mediated rejection; (a)HUS, (atypical) hemolytic uremic syndrome; BWG, Banff working group; Clin, clinical data; CNI, calcineurin inhibitor; EM, electron microscopy; #D, differential diagnosis; DIC, disseminated intravascular coagulation; EC, endothelial cells; Gen, genetic criterion; GN, glomerulonephritis; HTN, hypertension; ICC, intraclass correlation; IF/IHC, immunofluorescence microscopy/immunohistochemistry; IgAN, IgA nephropathy; Lab, laboratory; LDH, lactate dehydrogenase; LM, light microscopy; MPGN, membranoproliferative GN; MDC, minimum diagnostic criteria; NT-ABMR, non-TMA-associated antibody-mediated rejection; PI GN, post-infectious glomerulonephritis; R, round; SLE, systemic lupus erythematosus; TTP, thrombotic thrombocytopenic purpura; TMA, thrombotic microangiopathy; Tx-TMA, transplant TMA; %A, percentage agreement; %AL, percentage agreement level.
