# Corrigendum: Thrombotic Microangiopathy in the Renal Allograft: Results of the TMA Banff Working Group Consensus on Pathologic Diagnostic Criteria

**DOI:** 10.3389/ti.2023.12047

**Published:** 2023-10-16

**Authors:** Marjan Afrouzian, Nicolas Kozakowski, Helen Liapis, Verena Broecker, Luon Truong, Carmen Avila-Casado, Heinz Regele, Surya Seshan, Josephine M. Ambruzs, Alton Brad Farris, David Buob, Praveen N. Chander, Lukman Cheraghvandi, Marian C. Clahsen-van Groningen, Stanley de Almeida Araujo, Dilek Ertoy Baydar, Mark Formby, Danica Galesic Ljubanovic, Loren Herrera Hernandez, Eva Honsova, Nasreen Mohamed, Yasemin Ozluk, Marion Rabant, Virginie Royal, Heather L. Stevenson, Maria Fernanda Toniolo, Diana Taheri

**Affiliations:** ^1^ Department of Pathology, John Sealy School of Medicine, University of Texas Medical Branch at Galveston, Galveston, TX, United States; ^2^ Department of Pathology, Medical University of Vienna, Vienna, Austria; ^3^ Nephrology Center, Ludwig Maximilian University of Munich, Munich, Germany; ^4^ Department of Pathology, Sahlgrenska University Hospital, Gothenburg, Sweden; ^5^ Department of Pathology and Genomic Medicine, The Houston Methodist Hospital, Houston, TX, United States; ^6^ Laboratory Medicine Program, Toronto General Hospital, University Health Network (UHN), Toronto, ON, Canada; ^7^ Department of Pathology and Laboratory Medicine, Weill Cornell Medicine, New York, NY, United States; ^8^ Arkana Laboratories, Little Rock, AR, United States; ^9^ Department of Pathology and Laboratory Medicine, Emory University, Atlanta, GA, United States; ^10^ Department of Pathology, Université de Sorbonne, Assistance Publique—Hôpitaux de Paris, Hôpital Tenon, Paris, France; ^11^ New York Medical College, Valhalla, NY, United States; ^12^ Department of Pathology and Immunology, Baylor College of Medicine, Houston, TX, United States; ^13^ Department of Pathology and Clinical Bioinformatics, Erasmus University Center Rotterdam, Rotterdam, Netherlands; ^14^ Institute of Experimental Medicine and Systems Biology, RWTH Aachen University, Aachen, Germany; ^15^ Departamento de Patologia Geral, Instituto de Ciências Biológicas, Universidade Federal de Minas Gerais, Belo Horizonte, Brazil; ^16^ Department of Pathology, Koç University School of Medicine, Istanbul, Türkiye; ^17^ Department of Anatomical Pathology, NSW Health Pathology, Callaghan, NSW, Australia; ^18^ School of Medicine and Public Health, College of Health, Medicine and Wellbeing, The University of Newcastle, Callaghan, NSW, Australia; ^19^ Department of Pathology, School of Medicine, University of Zagreb, Zagreb, Croatia; ^20^ Department of Pathology and Laboratory Medicine, Mayo Clinic, Rochester, MN, United States; ^21^ AeskuLab Pathology and Department of Pathology, Charles University, Prague, Czechia; ^22^ Department of Pathology and Laboratory Medicine, King Fahad Specialist Hospital-Dammam, Dammam, Saudi Arabia; ^23^ Department of Pathology, Istanbul Faculty of Medicine, Istanbul University, Istanbul, Türkiye; ^24^ Department of Pathology, Necker-Enfants Malades Hospital, Université Paris Cité, Paris, France; ^25^ Department of Pathology, Maisonneuve-Rosemont Hospital, University of Montreal, Montreal, QC, Canada; ^26^ Kidney Pancreas Transplantation, Instituto de Nefrología-Nephrology, Buenos Aires, Argentina; ^27^ Department of Pathology, Isfahan Kidney Diseases Research Center, Isfahan University of Medical Sciences, Isfahan, Iran; ^28^ Urology Research Center, Sina Hospital, Tehran University of Medical Sciences, Tehran, Iran

**Keywords:** thrombotic microangiopathy, kidney, transplant, pathology criteria, Delphi, Banff

In the published article, there was an error with affiliations 3 and 4. Instead of “^3^Department of Pathology and Immunology, School of Medicine, Washington University in St. Louis, St. Louis, MO, United States”, and “^4^Geo-Bio Center, Ludwig Maximilian University of Munich, Munich, Germany”, it should be “^3^Nephrology Center, Ludwig Maximilian University of Munich, Munich, Germany”. The remaining affiliations have been renumbered.

There was an error in affiliation 4. Instead of “^4^Department of Clinical Pathology, Sahigrenska University Hospital, Gothenburg, Sweden” it should be “^4^Department of Pathology, Sahlgrenska University Hospital, Gothenburg, Sweden”.

There was an error in affiliation 5. Instead of “^5^Department of Pathology, Houston Methodist Hospital, Houston, TX, United States” it should be “^5^Department of Pathology and Genomic Medicine, The Houston Methodist Hospital, Houston, TX, United States”.

There was an error in affiliation 6. Instead of “^6^Laboratory Medicine Program, University Health Network (UHN), Toronto, ON, Canada” it should be “^6^Laboratory Medicine Program, Toronto General Hospital, University Health Network (UHN), Toronto, ON, Canada”.

There was an error in affiliation 13. Instead of “^13^Department of Pathology and Clinical Bioinformatics, Erasmus MC Transplant Institute, University Medical Center Rotterdam, Rotterdam, Netherlands” it should be “^13^Department of Pathology and Clinical Bioinformatics, Erasmus University Center Rotterdam, Rotterdam, Netherlands”.

There was an error in affiliation 16. Instead of “^16^Department of Pathology, School of Medicine, Koç University, Istanbul, Türkiye” it should be “^16^Department of Pathology, Koç University School of Medicine, Istanbul, Türkiye”.

There was an error in affiliation 18. Instead of “^18^School of Medicine and Public Health, University of Newcastle, Callaghan, NSW, Australia” it should be “^18^School of Medicine and Public Health, College of Health, Medicine and Wellbeing, The University of Newcastle, Callaghan, NSW, Australia”.

There was an error in affiliation 15. Instead of “^15^Departamento de Parasitologia, Instituto de Ciências Biológicas, Universidade Federal de Minas Gerais” it should be “^15^Departamento de Patologia Geral, Instituto de Ciências Biológicas, Universidade Federal de Minas Gerais, Belo Horizonte, Brazil”.

There was an error regarding the affiliation for Diana Taheri. As well as having affiliation “^27^Department of Pathology, Isfahan Kidney Diseases Research Center, Isfahan University of Medical Sciences, Isfahan, Iran”, they should also have “^28^Urology Research Center, Sina Hospital, Tehran University of Medical Sciences, Tehran, Iran”.

There was an error in the **Acknowledgments** segment of the article, which originally read “The authors would like to acknowledge the support of Dr. Reza Alaghehbandan for statistical analysis of R5 data, Dr. Michael Mengel for helpful discussions and suggestions, and Dr. Jan Ulrich Becker for useful discussions in the initial steps of the project.”

As the corresponding author had not obtained a written approval from Dr. Jan Ulrich Becker for this acknowledgment, at his request, his name is being removed from the **Acknowledgments** section.

The updated **Acknowledgments** section reads “The authors would like to acknowledge the support of Dr. Reza Alaghehbandan for statistical analysis of R5 data, and Dr. Michael Mengel for helpful discussions and suggestions.”

The authors apologize for all the above-mentioned errors and state that these errors and corrections do not change the scientific conclusions of the article in any way. The original article has been updated.

