# Supplementary material for: Thrombotic Microangiopathy in the Renal Allograft: Results of the TMA Banff Working Group Consensus on Pathologic Diagnostic Criteria
Source: Transpl Int. 2023 Aug 23;36:11590. doi: 10.3389/ti.2023.11590 (PMC10481335; doi:10.3389/ti.2023.11590)
Supplement: Supplementary file 1 [file Table1.DOCX]

**Table S1. Questions asked during R0.**

| Questions |
| --- |
| 1. What is the estimated percentage of diagnosis of Tx-TMA in your service? |
| 1. Have you seen Tx-TMA and AMR together? |
| 1. Have you seen Tx-TMA and TCMR together? |
| 1. Have you seen Tx-TMA and TG together? |
| 1. What stains do you use to make the diagnosis of Tx-TMA by LM? |
| 1. In your practice, which one of the following options is used in the diagnosis/ confirmation of TMA in renal allografts? |
| 1. Would you be able to contribute to the study by sending cases of recurrent/ de novo TMA? |
| 1. Light microscopic criteria for diagnosis of Tx-TMA (acute/ organizing) in the transplant kidney should include presence of: |
| 1. Electron microscopic criteria for diagnosis of Tx-TMA should include which of the following? |
| 1. Which of the following diagnostic steps are taken to establish the etiology of recurrent Tx-TMA, in your institution? |
| 1. Which of the following clinical, laboratory and histologic findings may help establish the diagnosis of recurrent TMA in your institution? |
| 1. In your practice, which modality (IHC/IF) do you prefer to use to assess the role of complement in transplant Tx-TMA? |
| 1. Which antibodies would you prefer to use to assess the role of complement in Tx-TMA? |
| 1. How often do you see Tx-TMA in your donor biopsies? |
| 1. Do you think the graft outcome is affected when Tx-TMA is seen in the donor biopsy? |
| 1. Does the donor type (non-related vs. related) make a difference in the outcome when a donor biopsy displays TMA? |
| 1. Do you have any Tx-TMA cases treated with Eculizumab? |
| 1. If yes, do you have a renal biopsy before and after Eculizumab treatment? |
| 1. Would you be able to send paraffin blocks of your Tx-TMA cases to be included in the study, to measure sets of endothelial cell gene transcripts? |
| 1. Would you be able to measure a set of endothelial cell gene transcripts on paraffin blocks of renal allograft biopsies at your institution? |
