# Supplementary material for: Thrombotic Microangiopathy in the Renal Allograft: Results of the TMA Banff Working Group Consensus on Pathologic Diagnostic Criteria
Source: Transpl Int. 2023 Aug 23;36:11590. doi: 10.3389/ti.2023.11590 (PMC10481335; doi:10.3389/ti.2023.11590)
Supplement: Supplementary file 2 [file Table2.DOCX]

**Table S2. Diagnosis on the original 37 cases and demographics.**

| Cases | Original diagnoses | Sex | Age |
| --- | --- | --- | --- |
| 1 | TMA | F | 22 |
| 2 | TMA (focal) + ABMR | M | 51 |
| 3 | TMA (acute and chronic) | F | 62 |
| 4 | TMA (classical case) | F | 44 |
| 5 | TMA (classical case) | M | 69 |
| 6 | TMA (Early) | M | 31 |
| 7 | TMA found on EM only | F | 5 |
| 8 | TMA found on EM only | M | 45 |
| 9 | TMA (classical case) | F | 45 |
| 10 | ABMR + TMA | M | 38 |
| 11 | TMA (classical case) | M | 35 |
| 12 | No TMA (suspicious for ABMR) | M | 40 |
| 13 | No TMA (TCMR + C4d-neg ABMR) | M | 72 |
| 14 | Subtle TMA + CNI tox | F | 39 |
| 15 | TMA (classical case) | M | 55 |
| 16 | TMA (classical case) | M | 55 |
| 17 | TMA with rare thrombi | M | 54 |
| 18 | TMA with small thrombi | M | 53 |
| 19 | No TMA (GN with deposits) | M | 63 |
| 20 | TMA (acute and chronic) | M | 45 |
| 21 | TMA (acute and chronic) | M | 38 |
| 22 | TMA + Nephrosclerosis | F | 48 |
| 23 | No TMA (Chronic ABMR + TG + weak C4d+) | F | 37 |
| 24 | No TMA (Chronic ABMR + TG + weak C4d+) | F | 44 |
| 25 | TMA (classical case) | F | 64 |
| 26 | TMA (classical case) | F | 60 |
| 27 | TMA + Hypertensive arteriopathy | M | 37 |
| 28 | TMA (classical case) | F | 60 |
| 29 | TCMR | F | 57 |
| 30 | TMA (focal) + ABMR | F | 31 |
| 31 | TMA (classical case) | M | 32 |
| 32 | No TMA | F | 54 |
| 33 | TMA (classical case) | F | 41 |
| 34 | No TMA (recurrent MPGN) | F | 52 |
| 35 | No TMA (recurrent IgA glomerulopathy) | M | 32 |
| 36 | TMA (classical case) | F | 30 |
| 37 | TMA + ABMR | M | 42 |

Note: Cases of “diffuse TMA” and “classical TMA” were those cases in which the diagnosis of TMA was easily made due to either presence of numerous glomeruli or vessels containing thrombi, or there was a point in patient’s history or laboratory results that had pointed towards a diagnosis of TMA. Cases of “focal TMA”, “early TMA” and TMA associated with other lesions such as ABMR were cases in which the nephropathologist had encountered significant difficulty in diagnosing Tx-TMA and help was obtained from EM. Cases of “TMA on EM only” were cases in which TMA was not present in LM, and TMA was found only on EM. Cases of “chronic TMA” were cases in which double contour and/or mesangial interposition were seen. Cases of Non-TMA were “Look-alike” cases in which the diagnosis of TMA was entertained during differential diagnosis.
