# Supplementary material for: Thrombotic Microangiopathy in the Renal Allograft: Results of the TMA Banff Working Group Consensus on Pathologic Diagnostic Criteria
Source: Transpl Int. 2023 Aug 23;36:11590. doi: 10.3389/ti.2023.11590 (PMC10481335; doi:10.3389/ti.2023.11590)
Supplement: Supplementary file 3 [file Table3.DOCX]

**Supplemental table S3. Literature review on TX-TMA.**

| Criteria | Authors | Year/ISO country code | Number of reported total allograft recipient population/ biopsy specimens in the centre(s) | Number of patients/ biopsy specimens with Tx-TMA studied | Maximal number of the specific lesion (=criterion) | Incidence of Tx-TMA in the total allograft recipient population (in %) | Incidence of Tx-TMA per biopsy (in %) | Prevalence of criterion in total allograft recipient population (in %) | Prevalence of criterion in total allograft biopsy specimens (in %) | Prevalence of criterion in the studied Tx-TMA population (in %) | Prevalence of criterion in the studied Tx-TMA biopsy specimens (in %) |  |
| --- | --- | --- | --- | --- | --- | --- | --- | --- | --- | --- | --- | --- |
| 1A. bloodless, dilated, congested glomerular capillaries | Broecker, V et al.^23^ | 2019/GB | -/- | 81/105 | 32 |  |  |  |  | 39,51 | 30,48 |  |
| 1B. fibrin thrombi in arterioles/ small arteries ± fibrinoid change | Afzal, F et al.11 | 2001/US | 629/- | 29/- |  | 4,61 |  |  |  |  |  |  |
|  | Nickelheit, V et al.^12^ | 2002/CH | -/398 | 30/30 |  |  | 7,54 |  |  |  |  |  |
|  | Reynolds, JC et al.^13^ | 2003/US | 15848/- | 127/- |  | 0,80 |  |  |  |  |  |  |
|  | Fortin, MC et al.^14^ | 2004/CA | 368/464 | 13/- |  | 3,53 | 2,80 |  |  |  |  |  |
|  | § Stolyarevich, ES et al.^15^ | 2006/RU | 230/- | 21/- |  | 9,13 |  |  |  |  |  |  |
|  | Satoskar, AA et al.^17^ | 2010/US | 958/1551 | 59/- | 34 | 6,16 | 3,80 | 3,55 | 2,19 | 57,63 |  | a |
|  | ✢ Meehan, SM et al.^18^ | 2011/US | -/307 | 14/14 | 7 |  | 4,56 |  | 2,28 | 50,00 |  | b |
|  | Meehan, SM et al.^18^ | 2011/US | 563/1073 | 37/37 |  | 6,57 | 3,45 |  |  |  |  | b |
|  | Sreedharanunni, S et al.^20^ | 2013/IN | -/1792 | 114/114 | 91 |  | 6,36 |  | 5,08 | 79,82 | 79,82 |  |
|  | § Gumber, M et al.^19^ | 2014/IN | 1175/1538 | 34/- |  | 2,89 | 2,21 |  |  |  |  |  |
|  | Wu, K et al.^22^ | 2016/DE | -/- | 62/62 | 32 |  |  |  |  | 51,61 | 51,61 | c |
|  | Broecker, V et al.^23^ | 2019/GB | -/- | 81/104 | 58 |  |  |  |  | 71,60 | 55,77 |  |
|  | Teixeira, CM et al.^25^ | 2020/BR | 9541/6886 | 89/89 | 45 | 0,93 | 1,50 | 0,47 | 0,65 | 50,56 | 50,56 | d |
|  | § Futamura,K et al.^13^ | 2020/JP | 1336/5425 | 69/- |  | 5,16 | 1,27 |  |  |  |  |  |
|  | Prokopenko, EI et al.^24^ | 2020/RU | 697/728 | 32/- |  | 4,59 | 4,40 |  |  |  |  |  |
| 1C. fibrin thrombi in glomerular capillaries / hilum | Satoskar, AA et al.^17^ | 2010/US | 958/1551 | 59/- | 59 | 6,16 | 3,80 | 6,16 | 3,80 | 100,00 |  |  |
|  | ✢ Meehan, SM et al.^18^ | 2011/US | -/307 | 14/14 | 10 |  | 4,56 |  | 3,26 | 71,43 | 71,43 |  |
|  | Wu, K et al.^22^ | 2016/DE | -/- | 62/62 | 35 |  |  |  |  | 56,45 | 56,45 |  |
|  | Broecker, V et al.^23^ | 2019/GB | -/- | 81/108 | 75 |  |  |  |  | 92,59 | 69,44 |  |
|  | Teixeira, CM et al.^25^ | 2020/BR | 9541/6886 | 89/89 | 63 | 0,93 | 1,29 | 0,66 | 0,91 | 70,79 | 70,79 | d |
| 1D. arterial or arteriolar intimal oedema/mucoid changes | Satoskar, AA et al.^17^ | 2010/US | 958/1551 | 59/- |  | 6,16 | 3,80 |  |  |  |  | a |
|  | ✢ Meehan, SM et al.^18^ | 2011/US | -/307 | 14/14 | 7 |  | 4,56 |  | 2,28 | 50,00 | 50,00 | b |
|  | Broecker, V et al.^23^ | 2019/GB | -/- | 81/104 | 47 |  |  |  |  | 58,02 | 45,19 |  |
| 1E. glomerular endothelial swelling | Teixeira, CM et al.^25^ | 2020/BR | 9541/6886 | 89/89 | 54 | 0,93 | 1,29 | 0,57 | 0,78 | 60,67 | 60,67 | d |
| 1F. mesangiolysis | ✢ Meehan, SM et al.^18^ | 2011/US | -/307 | 14/14 | 2 |  | 4,56 |  | 0,65 | 14,29 | 14,29 | b |
|  | Meehan, SM et al.^18^ | 2011/US | 563 | 37/37 |  |  | 3,45 |  |  |  |  | b |
|  | Broecker, V et al.^23^ | 2019/GB | -/- | 81/108 | 15 |  |  |  |  | 18,52 | 13,89 |  |
|  | Teixeira, CM et al^12^ | 2020/BR | 9541/6886 | 89/89 | 28 | 0,93 | 1,29 | 0,29 | 0,41 | 31,46 | 31,46 | d |
| 1G. double contours | Satoskar, AA et al.^17^ | 2010/US | 958/1551 | 59/- |  | 6,16 | 3,80 |  |  |  |  | a |
|  | ✢ Meehan, SM et al.^18^ | 2011/US | -/307 | 14/14 | 5 |  | 4,56 |  | 1,63 | 35,71 | 35,71 | b |
|  | Wu, K et al.^22^ | 2016/DE | -/- | 62/62 | 29 |  |  |  |  | 46,77 | 46,77 | c |
|  | Broecker, V et al. ^23^ | 2019/GB | -/- | 81/105 | 10 |  |  |  |  | 12,35 | 9,52 |  |
| 1H. platelet thrombi in glomerular capillaries | Meehan, SM et al.^16^ | 2008/US | -/- | 28/28 | 23 |  |  |  |  | 82,14 | 82,14 |  |
| 1I. fragmented / extravasated RBCs | Broecker, V et al.^23^ | 2019/GB | -/- | 81/105 | 24 |  |  |  |  | 29,63 | 22,86 |  |
| 1K. collapsed capillaries | ✢ Meehan, SM et al.^18^ | 2011/US | -/307 | 14/14 | 12 |  | 4,56 |  | 3,91 | 85,71 | 85,71 | b |
|  | Broecker, V et al.^23^ | 2019/GB | -/- | 81/105 | 31 |  |  |  |  | 38,27 | 29,52 |  |
| 4A. C4d positivity (favouring AMR vs TMA) | Satoskar, AA et al.^17^ | 2010/US | 958/1551 | 59/- | 33 | 6,16 | 3,80 | 3,44 | 2,13 | 55,93 |  | a |
|  | Meehan, SM et al.^18^ | 2011/US | 563 | 37/37 | 6 | 6,57 | 3,45 | 1,07 | 0,56 | 16,22 | 16,22 | b |
|  | Sreedharanunni, S et al.^20^ | 2013/IN | -/1792 | 114/114 | 50 |  | 6,36 |  | 2,79 | 43,86 | 43,86 |  |
|  | Chua, JS et al.^21^ | 2015/NL | -/- | 12/- | 12 |  |  |  |  | 100,00 |  |  |
|  | Wu, K et al.^22^ | 2016/DE | -/- | 62/62 | 12 |  |  |  |  | 19,35 | 19,35 | c |
|  | Teixeira, CM et al.^25^ | 2020/BR | 9541/6886 | 89/89 | 13 | 0,93 | 1,29 | 0,14 | 0,19 | 14,61 | 14,61 | d |
| 5. EM+ | Sreedharanunni, S et al.^20^ | 2013/IN | -/1792 | 114/114 | 14 |  | 6,36 |  | 0,45 | n.a. |  | f |
|  | Broecker, V et al.^23^ | 2019/GB | -/- | 81/108 | 49 |  |  |  |  | n.a. |  | e |

As shown here, to this date, most Tx-TMA-related publications was focused on clinical features that potentially could differentiate between the causes of TMA, such as calcineurin-inhibitor toxicity, ABMR and recurrence of disease-associated Tx-TMA. Others have tried to correlate pathologic findings with graft outcome. For instance, a study published in 2011 showed more severe lesions in C4d-positive Tx-TMA (consistent with ABMR) and potentially worse prognosis than Tx-TMA in C4d-negative biopsies.

Abbreviations: ABMR: antibody-mediated rejection, EM: electron microscopy, EM+: electron microscopy positive, LM: light microscopy, RBC: red blood cell, TMA: thrombotic microangiopathy.

✢ in the cited manuscript both lesions in early biopsies or in the whole cohort were reported, ✢ means when only early biopsies were taken into consideration.

§ data from poster.
